# Mesenchymal Stem Cell-Conditioned Media-Loaded Microparticles Enhance Acute Patency in Silk-Based Vascular Grafts

**DOI:** 10.3390/bioengineering11090947

**Published:** 2024-09-21

**Authors:** Katherine L. Lorentz, Ande X. Marini, Liza A. Bruk, Prerak Gupta, Biman B. Mandal, Morgan V. DiLeo, Justin S. Weinbaum, Steven R. Little, David A. Vorp

**Affiliations:** 1 Department of Bioengineering, University of Pittsburgh, Pittsburgh, PA 15261, USAande.marini@pitt.edu (A.X.M.); lab154@pitt.edu (L.A.B.); morgandileo@pitt.edu (M.V.D.); juw51@pitt.edu (J.S.W.); srlittle@pitt.edu (S.R.L.); 2 Department of Biosciences and Bioengineering, Indian Institute of Technology Guwahati, Guwahati 781039, Assam, India; biman.mandal@iitg.ac.in; 3 Centre for Nanotechnology, Indian Institute of Technology Guwahati, Guwahati 781039, Assam, India; 4 Jyoti and Bhupat Mehta School of Health Sciences and Technology, Indian Institute of Technology Guwahati, Guwahati 781039, Assam, India; 5 Department of Ophthalmology, University of Pittsburgh, Pittsburgh, PA 15213, USA; 6 McGowan Institute for Regenerative Medicine, University of Pittsburgh, Pittsburgh, PA 15219, USA; 7 Department of Chemical and Petroleum Engineering, University of Pittsburgh, PA 15261, USA; 8 Clinical & Translational Sciences Institute, University of Pittsburgh, Pittsburgh, PA 15213, USA; 9 Department of Pathology, University of Pittsburgh, Pittsburgh, PA 15260, USA; 10 Department of Immunology, University of Pittsburgh, Pittsburgh, PA 15213, USA; 11 Department of Pharmaceutical Sciences, University of Pittsburgh, Pittsburgh, PA 15213, USA; 12 Department of Surgery, University of Pittsburgh, Pittsburgh, PA 15213, USA; 13 Department of Mechanical Engineering and Materials Science, University of Pittsburgh, PA 15261, USA; 14 Department of Cardiothoracic Surgery, University of Pittsburgh, Pittsburgh, PA 15213, USA; 15 Magee Women’s Research Institute, Pittsburgh, PA 15213, USA

**Keywords:** tissue-engineered vascular graft, mesenchymal stem cells, coronary artery disease, cardiovascular disease, regenerative medicine

## Abstract

Coronary artery disease leads to over 360,000 deaths annually in the United States, and off-the-shelf bypass graft options are currently limited and/or have high failure rates. Tissue-engineered vascular grafts (TEVGs) present an attractive option, though the promising mesenchymal stem cell (MSC)-based implants face uncertain regulatory pathways. In this study, “artificial MSCs” (ArtMSCs) were fabricated by encapsulating MSC-conditioned media (CM) in poly(lactic-co-glycolic acid) microparticles. ArtMSCs and control microparticles (Blank-MPs) were incubated over 7 days to assess the release of total protein and the vascular endothelial growth factor (VEGF-A); releasates were also assessed for cytotoxicity and promotion of smooth muscle cell (SMC) proliferation. Each MP type was loaded in previously published “lyogel” silk scaffolds and implanted as interposition grafts in Lewis rats for 1 or 8 weeks. Explanted grafts were assessed for patency and cell content. ArtMSCs had a burst release of protein and VEGF-A. CM increased proliferation in SMCs, but not after encapsulation. TEVG explants after 1 week had significantly higher patency rates with ArtMSCs compared to Blank-MPs, but similar to unseeded lyogel grafts. ArtMSC explants had lower numbers of infiltrating macrophages compared to Blank-MP explants, suggesting a modulation of inflammatory response by the ArtMSCs. TEVG explants after 8 weeks showed no significant difference in patency among the three groups. The ArtMSC explants showed higher numbers of SMCs and endothelial cells within the neotissue layer of the graft compared to Blank-MP explants. In sum, while the ArtMSCs had positive effects acutely, efficacy was lost in the longer term; therefore, further optimization is needed.

## 1. Introduction

Cardiovascular disease is the current leading cause of death globally [1,2]. Coronary artery disease is the most common form of cardiovascular disease, leading to over 360,000 mortalities in 2019 in the United States [1,3]. Occlusion in the coronary artery is typically bypassed by a graft, where the current standards are either the autologous internal mammary artery or saphenous vein. However, both of these autologous options are limited [4,5]. The saphenous vein has also been known to have high long-term failure rates [5]. Thus, tissue-engineered vascular grafts (TEVGs) have been sought as potential alternatives by our group [6,7,8,9,10,11] and others [12,13,14,15,16,17,18,19,20,21,22,23,24].

In order for TEVGs to be successful, they must have low immunogenicity in regard to both their scaffold material and their bioactive cargo [25]. Silk has been utilized in various tissue-engineering applications due to its tunable biodegradation, low immunogenicity, and overall biocompatibility [7,26]. Some tissue-engineered products containing silk have gained FDA approval [26,27]. Mesenchymal stem cells (MSCs) have been shown to be immune evasive and anti-inflammatory and have shown promise for improved tissue-engineering applications through their regenerative properties [28,29]. MSC-conditioned media (CM), which contains the MSC secretome, has shown promise as a therapeutic in cardiovascular applications [30,31]. This secretome includes growth factors such as vascular endothelial growth factor (VEGF), hepatocyte growth factor, fibroblast growth factor, and transforming growth factor β [32,33], which are known for their angiogenic effects. Additionally, MSC secretome has been shown to reduce interferon γ and tissue necrosis factor α secretion from inflammatory cells [34], thus making the MSC secretome an attractive therapeutic. In this work, we propose that CM can be used to generate “artificial MSCs.” It is a valid concern that calling our technology “artificial MSCs” (or ArtMSCs for short) is a strong assumption, as they do not proliferate or actively respond to their environment like cells. However, this is the term we used in patenting the technology (US20230285290A1), so we would prefer to continue using it here.

Our lab has previously reported success with MSC-seeded TEVG constructs [8,9,11] compared to unseeded controls. However, the inclusion of MSCs in a TEVG presents various regulatory barriers and, hence, clinical acceptance. Therefore, a cell-free alternative to mitigate these possible risks is warranted. Our lab and others have shown that secreted factors from MSCs in conditioned media can have regenerative effects on other cells [35,36,37,38]. Utilizing these secreted factors to harness the complex immunoregulatory and regenerative properties of the MSCs without using the original cells could provide the cargo for a cell-based, but cell-free, TEVG [39].

We have previously shown that degradable microparticles (MPs) encapsulated with C-C motif chemokine ligand 2 (CCL2, also known as monocyte chemoattractant protein-1) improved patency for silk-based TEVGs after initial success at 1 week of implantation [10]. However, longer-term studies at 8 weeks did not see desirable patency rates (54% (7/13)). As MSCs contain many regenerative and other factors within their conditioned media, the hypothesis of this study was that this MSC secretome could extend remodeling past what had been achieved with CCL2 alone and provide long-term patency.

This study comprised in vitro experiments to investigate the secreted factors from MSCs loaded in PLGA microparticles (and as it happened, their deficiencies) and in vivo testing of silk+ArtMSC TEVGs. For in vitro characterization of the ArtMSCs, release assays were used to observe their release kinetics in addition to determining whether two specific factors hypothesized to be important for TEVG success—vascular endothelial growth factor A (VEGF-A) and urokinase plasminogen activator (uPA)—were present in the releasates. Implantations were performed as abdominal aortic interposition grafts for 1 or 8 weeks in Lewis rats with patency assessed at explant, as well as further characterization of TEVG composition. The combined analysis of in vivo and in vitro activity was performed to obtain a robust understanding of the regenerative potential of this delivery system.

## 2. Materials and Methods

### 2.1. Conditioned Media (CM) Generation

The CM was generated from human adipose-derived mesenchymal stem cells (hADMSCs) (RoosterVial-hAD-1M MSC Lot #00097, RoosterBio, Frederick, MD, USA). A stock vial of 1 million cells at passage 2 was thawed into a T175 flask (Nunc, ThermoFisher Scientific, Pittsburgh, PA, USA) and cultured in 20 mL of RoosterNourish growth media (GM) (KT-001, RoosterBio) for 3 days. The hADMSCs were then passaged at 1.5 million cells per T175 flask and cultured in GM to 40% confluency, at which point they were washed with 1X Hank’s buffered saline solution (HBSS, pH 7.4, Gibco, Gaithersburg, MD, USA) and 25 mL of harvest media was applied. Harvest media consisted of DMEM (21063029, Gibco), 5% FBS (Atlanta Biologics, Flowery Branch, GA, USA), 1% penicillin streptomycin (Gibco), 0.1% Amphotericin B (Gibco), and 10 µL/L of dexamethasone (1126, Tocris, Minneapolis, MN, USA). After 48 h of culture, the CM was removed and centrifuged (Sorvall Legend RT Centrifuge, Pittsburgh, PA, USA) at 2040× *g* at 4 °C for 5 min to remove any dead cells or large debris. Following centrifugation, the supernatant was stored at −80 °C until use.

### 2.2. CM Microparticle (ArtMSC) Fabrication

For fabrication of the ArtMSCs, 20 mL aliquots of CM (MSC secretome) was filtered using 10 kDa filter tubes (Amicon Ultra, Millipore Sigma, Burlington, MA, USA), lyophilized overnight, and resuspended in 450 µL of DI water at a 10x concentration as described in [33]. Concentrated CM was encapsulated using a water–oil–water double emulsion procedure in poly(lactic-co-glycolic acid) (PLGA) MPs, as previously described [10,33,40]. The oil solution was formed by dissolving PLGA (200 mg, 739952-5G, Millipore Sigma) in 4 mL of dichloromethane. This solution was sonicated for 10 s at 30% amplitude (EpiShear Probe Sonicator, Active Motif, Carlsbad, CA, USA) and then homogenized in a 2% polyvinyl alcohol (PVA) solution (60 mL) for 1 min at 7000 rpm (Silverson L5M-A, East Longmeadow, MA, USA) to form PLGA droplets in an aqueous phase. After homogenization, the solution was stirred for 3 h at 600 rpm in a 1% PVA solution to allow for evaporation of organic solvent, forming solid MPs. MPs were then washed 4 times using centrifugation. The washed MPs were decanted to minimize residual water, flash frozen in liquid nitrogen, and then lyophilized for a minimum of 48 h. This same process was also used to fabricate Blank MPs (Blank-MPs) to analyze the effects of PLGA, replacing the ultrapure water for the CM. Three batches of ArtMSCs and one batch of Blank-MPs were used for each round of analysis.

### 2.3. Characterization of CM and ArtMSC Morphology and Release

Analysis of uPA activity in one batch of CM was performed with a Urokinase Activity Assay (AS-72159, AnaSpec, Fremont, CA, USA), according to the manufacturer’s protocols, with non-conditioned media (GM without being exposed to cells, nCM) as a control. The morphology of the ArtMSCs was qualitatively characterized using SEM (JEOL JSM 6335F, Peabody, MA, USA). The diameter was then quantified with volume impedance measurements (n = 10,000 MPs per sample) (Multisizer, Beckman Coulter, Brea, CA, USA), and microparticle density was measured using the final compressed volume of a known mass of MPs.

To measure protein release, ArtMSCs or Blank-MPs were added (5 mg) to 500 µL of phosphate-buffered saline (PBS, pH 7.4, Gibco) in triplicate in 1.5 mL microcentrifuge tubes (ThermoFisher Scientific) and placed in an end-over-end turner at 37 °C. A release assay was performed over 7 days, collecting releasates every 6 h for the first 24 h and then every 24 h after that. Releasates were centrifuged at 10,000× *g* at 4 °C for 5 min (5415R Centrifuge, Eppendorf) to pellet MPs. Supernatant was removed and stored at −80 °C until ready for analysis.

A micro bicinchoninic assay kit (BCA, Thermofisher) was used to measure the total protein released from MPs. Next, either 150 µL of each releasate or a standard curve (in triplicate) from 0 to 200 µg of bovine serum albumin diluted in PBS was added to 96-well plates, and 150 µL of working reagent was added to each well to cause the colorimetric reaction. The plate was incubated at 37 °C for 2 h and read at 562 nm with a microplate reader to measure absorbance to determine protein concentration (Synergy HT, BioTek Instruments, Vermont, MA, USA).

A DuoSet enzyme-linked immunosorbent assay (ELISA) Ancillary Reagent Kit (#DY008, R&D Systems, Minneapolis, MN, USA) and respective Quantikine ELISA Kits were used to measure VEGF-A (DY293B-05) and uPA (DY1310) release from MPs. For this, 100 µL of each releasate or a standard curve (in triplicate) of either VEGF-A (0 to 2000 pg/mL) or uPA (0 to 4000 pg/mL) diluted in reagent diluent (from Ancillary kit) was added to 96-well plates. Samples were analyzed according to the manufacturer’s protocols.

### 2.4. Vascular Cell Culture

Human aortic vascular smooth muscle cells (SMCs) either from ATCC (for toxicity assessment, Manassas, VA, PCS-100-012) or Cell Applications (for proliferation assays, 354-05a, San Diego, CA, USA) were cultured in supplemented basal media (SBM) (311K-500, Cell Applications Inc.). Human coronary artery endothelial cells (ECs) from Cell Applications (300-05a) were also cultured in supplemented basal media (212K-500, Cell Applications Inc.). SMCs and ECs were used between passages 6–10.

### 2.5. MP Toxicity Assessment

A LIVE/DEAD assay (R37601, ThermoFisher Scientific) was used to analyze the toxicity of the ArtMSC and Blank-MP releasates. A total of 3.0 mg of either ArtMSCs or Blank-MPs was added into 1 mL of PBS for 24 h of release. SMCs (ATCC) were plated at 40,000 cells/well into a 24-well plate. SMCs were incubated overnight in SMC SBM (Cell Applications Inc.). The media were removed and replaced with 500 µL of treatment (ArtMSC or Blank-MP releasates) for 12 h. SMC SBM (positive viability control) and diH_2_O (positive death control) were also applied to cells in triplicate to validate staining of live and dead cells. All wells were fluorescently imaged (Eclipse 90i and NIS Elements, Nikon, Tokyo, Japan). The number of live and dead cells were quantified using ImageJ 1.54j (National Institutes of Health, Bethesda, MD, USA). Images were split into red and green channels and the number of cells were counted in each channel using the ImageJ thresholding function. An optimal threshold for green and red cell counts was determined and used for counting the number of cells in all images.

### 2.6. SMC and EC Proliferation

Proliferation assays were performed based on a modified protocol [6]. Type 1 rat tail collagen (Advanced Biomatrix, NC1558174) was dissolved in 0.02 M acetic acid to a concentration of 50 µg/mL. Then 200 µL of collagen solution was added to each well of a 48-well plate and incubated at room temperature for 1 h. The wells were next washed 3 times with PBS and either SMCs or ECs (Cell Applications Inc.) were plated at 10,000 cells per well in 1 mL of respective SBM. Cells were cultured overnight prior to baseline reading of cellular activity. SBM was removed from the wells, and the wells were washed with PBS. Cellular activity was measured at baseline by adding 300 µL of respective unsupplemented basal media (BM) and 30 µL of alamar blue (Invitrogen, DAL1100) to each well and cultured at 37 °C and 5% CO_2_ for 3 h. Following incubation, 100 µL of solution from each well was added to a 96-well plate and read at 570 and 600 nm with a plate reader. The alamar blue solution was removed and the wells were washed with PBS. The following treatments were then added in a 1:1 ratio (150 µL BM to 150 µL treatment). Treatments consisted of the following: 3 different batches of ArtMSC releasates, Blank-MPs releasates, and the respective CM were loaded into the ArtMSCs, SBM (positive control), and BM (negative control). Each batch of CM used for fabrication of ArtMSCs and ArtMSC releasates was analyzed for its effect on SMC and EC proliferation as an individual batch and collectively as all three batches. ArtMSCs and Blank-MPs (7 mg) were released into respective BM (700 µL) for 24 h prior to adding to cells and centrifuged at 16,000× *g* at 4 °C for 5 min to pellet microparticles; supernatant was removed and used as treatment. After 24 h of culture in treatments, treatments were removed, wells were washed with PBS, and cellular activity was measured as described above. The difference in cellular activity following treatment was analyzed by normalizing to baseline cellular activity.

### 2.7. Fabrication of MP Lyogel Silk Constructs

**Figure 1:**
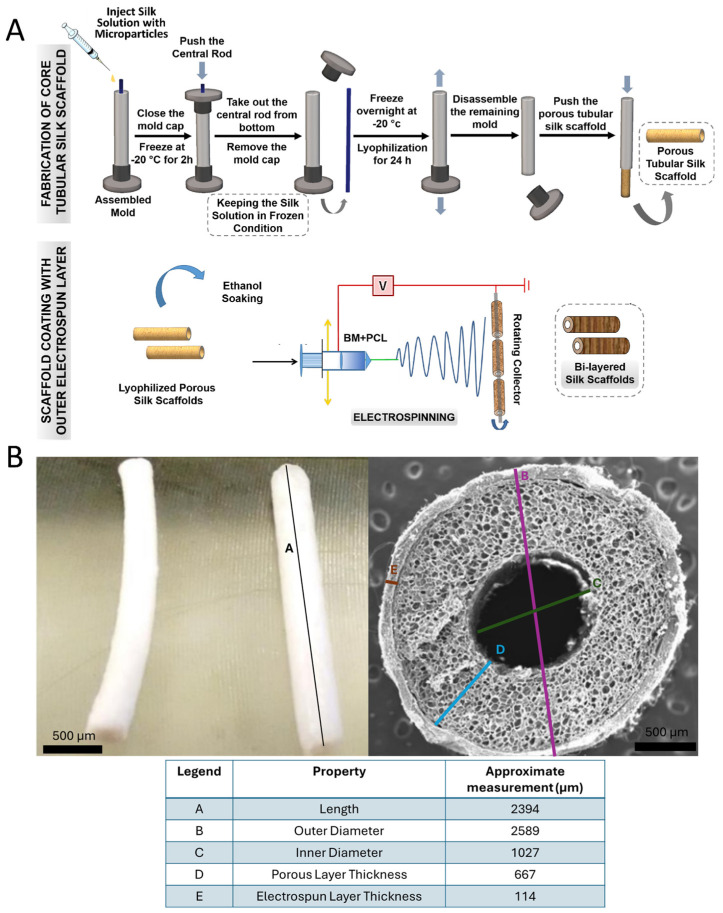
**Schematic demonstrating the fabrication of the lyogel scaffolds with and without microparticles** (**A**) **and representative image of unloaded lyogel scaffold** (**B**). Microparticles are mixed directly with the silk solution used to create the inner porous layer (**A**). The bi–layered scaffold consists of an inner porous layer that is fabricated through lyophilization within a metal rod and an outer nanofibrous layer that is created through an electrospinning procedure. The image is adapted from [7]. (This article was published in *Acta Biomaterialia*, 105, P. Gupta, K.L. Lorentz, D.G. Haskett, E.M. Cunnane, A.K. Ramaswamy, J.S. Weinbaum, D.A. Vorp, B.B. Mandal, Bioresorbable silk grafts for small diameter vascular tissue engineering applications: In vitro and in vivo functional analysis, 146–158, Copyright Elsevier (2020)). Five different properties of a representative scaffold are provided (length (A), outer diameter (B), inner diameter (C), porous layer thickness (D), and electrospun layer thickness (E)) (**B**). Scale bar = 500 µm.

Scaffolds were fabricated according to previously published protocols as outlined in [10] and as a schematic in Figure 1A [7]. (This article was published in *Acta Biomaterialia*, 105, P. Gupta, K.L. Lorentz, D.G. Haskett, E.M. Cunnane, A.K. Ramaswamy, J.S. Weinbaum, D.A. Vorp, B.B. Mandal, Bioresorbable silk grafts for small diameter vascular tissue engineering applications: In vitro and in vivo functional analysis, 146–158, Copyright Elsevier (2020)). In short, silk fibroin solution was generated from two sources: Bombyx mori (BM) cocoons and Antheraea assama (AA) glands. A total of 3 mg of either Blank-MPs or ArtMSCs was mixed into the lyogel silk solution. These solutions were mixed in a 1:1 ratio (6% BM and 2% AA) and injected into a scaffold mold (length: 3 cm, central rod diameter: 1.1 mm, and inner diameter of concentric outer cylinder: 2 mm). They were then incubated at 37 °C for initial gelation. Following incubation, the silk solution within the mold was frozen at −20 °C overnight and then lyophilized for 24 h to create a porous scaffold. This scaffold was referred to as lyogel, and then placed in 80% ethanol for 15 min after being removed from the mold. The scaffold was then electrospun with a 10% (*w*/*v*) polycaprolactone (Millipore Sigma) and 10% (*w*/*v*) BM silk in 11,1,3,3,3-Hexafluoro-2-propanol (Millipore Sigma) at a 1:1 ratio. Three scaffold groups were fabricated and tested: unloaded lyogel, Blank-MP-loaded lyogel, and ArtMSC-loaded lyogel. A schematic with approximate dimensions of an unloaded lyogel scaffold is shown in Figure 1B.

### 2.8. In Vivo Implantation of ArtMSC Lyogel Silk Constructs

All implants were performed according to a University of Pittsburgh Institutional Animal Care and Use Committee approved protocol, in compliance with the ARRIVE guidelines. A power analysis was conducted using data from [41] and mechanical testing as a stringent metric, with effect size of 1.59, alpha of 0.05, and power of 0.8. This resulted in a required group size of n = 3. Grafts were implanted as abdominal aortic interposition grafts in 3- to 6-month-old male Lewis rats (Charles River, Wilmington, MA, USA) according to previous protocols [6,10]. In brief, rats were anesthetized with isoflurane (1%) and ketamine (50 mg/kg) and then a midline incision was made on the abdomen. Following incision, a 1–1.5 cm portion of the aorta was isolated. Micro clamps (#10011-531 VascuStatt II, Scanlan, St. Paul, MN, USA) were used to stop blood flow to the isolated area. Following clamping, the aorta was cut in the middle of the clamped portion, causing a 1 cm gap from elastic recoil of the vessel. The 1 cm long ArtMSC lyogel silk construct was then inserted with end-to-end anastomoses using interrupted sutures (10-0 prolene, #2794G, eSutures, Mokena, IL, USA). Following the securing of the graft, clamps were removed to restore blood flow. The muscle and skin layers were then closed using running 3-0 polyglactin resorbable sutures (J215H, Ethicon, Somerville, NJ, USA). Both dipyridamole (250 mg/kg for the first 7 days, 100 mg/kg for the following 3 weeks) and aspirin (200 mg/kg for the first 7 days, 100 mg/kg for the following 2 weeks), two anticoagulants, were administered orally.

Patency was evaluated at both 1 week and 8 weeks in grafts consisting of the following groups: unloaded lyogel scaffolds (lyogel), Blank-MP-loaded lyogel constructs (Blank-MPs), and ArtMSC-loaded lyogel constructs (ArtMSCs). At 1 week or 8 weeks, the rats were anesthetized with isoflurane (2–5%) and then euthanized by a lethal intracardiac injection of heparin (40IU, McKesson Medical-Surgical, Livingston, MT, USA) and potassium chloride (2 mL/rat). Directly after being euthanized, a catheter (22GX1 in, Safelet IV catheter, Excel Inc., Seattle, WA, USA) was placed into the left ventricle and an angiogram (GE OEC 9800 Plus, G.E., Boston, MA, USA) was taken to assess TEVG patency. A contrast agent (ISOVUE, Bracco, Monroe Township, NJ, USA) was injected into the catheter to perform the angiogram. The TEVG was then explanted for histologic imaging.

### 2.9. Immunofluorescent Staining and Histochemical Analysis

Explants were prepared according to previous protocols [10]. The middle portions (3–5 mm) of the grafts were fixed in 2% paraformaldehyde for 1.5 h and then washed in PBS. These sections were then fixed and embedded in paraffin. The samples were then immunofluorescently (IFC) stained for three different cell types associated with remodeling of an implanted construct toward a TEVG. First, contractile vascular smooth muscle cells were identified using calponin (1:100 ab46794, Abcam, Cambridge, UK)/Cy5 (1:100 ab150075, Abcam) and α-smooth muscle actin (1:100 ab7817, Abcam)/Cy5 (α-SMC 1:100 ab234082, Jackson ImmunoResearch Laboratories, West Grove, PA). Endothelial cells were identified using the von Willebrand Factor (vWF) preconjugated with FITC (1:100 ab195028, Abcam). Macrophages were stained with CD68 (1:100 ab31630, monoclonal, Abcam)/Cy5 (1:100 ab234080, Jackson ImmunoResearch Laboratories). Histochemistry was performed to observe cellularity (via hematoxylin and eosin (H&E)) and elastic fiber formation (via Verhoeff–Van Gieson (VVG)).

A custom MATLAB code was used to quantify IFC staining of macrophages as previously described [10]. Images were manually segmented to define masks for the inner (porous) and outer (electrospun) layers, saved as files with anonymized names for blinding, and then manually thresholded for automated counting of cells in each layer in MATLAB R2020a.

### 2.10. Statistical Analysis

Statistical analyses were performed to determine significance between groups. Student’s *t*-tests were used to compare diameter and density. A two-way repeated measures ANOVA with a post hoc Tukey’s test was used for release assays. A one-way analysis of variance (ANOVA) with a post hoc Tukey’s test was used for proliferation assays and analysis of CD68+ cells between TEVG groups. A paired *t*-test was used to determine CD68+ cells differences between the inner and outer explant layers. A Fisher’s exact test was used to determine differences in patency. Significance was represented as a *p*-value less than 0.05. Results are reported as mean ± standard deviation (S.D.) and with n = 4 unless specified differently. All results were analyzed in Graphpad Prism 9 (GraphPad, San Diego, CA, USA).

## 3. Results

### 3.1. Characterization of ArtMSCs

**Figure 2:**
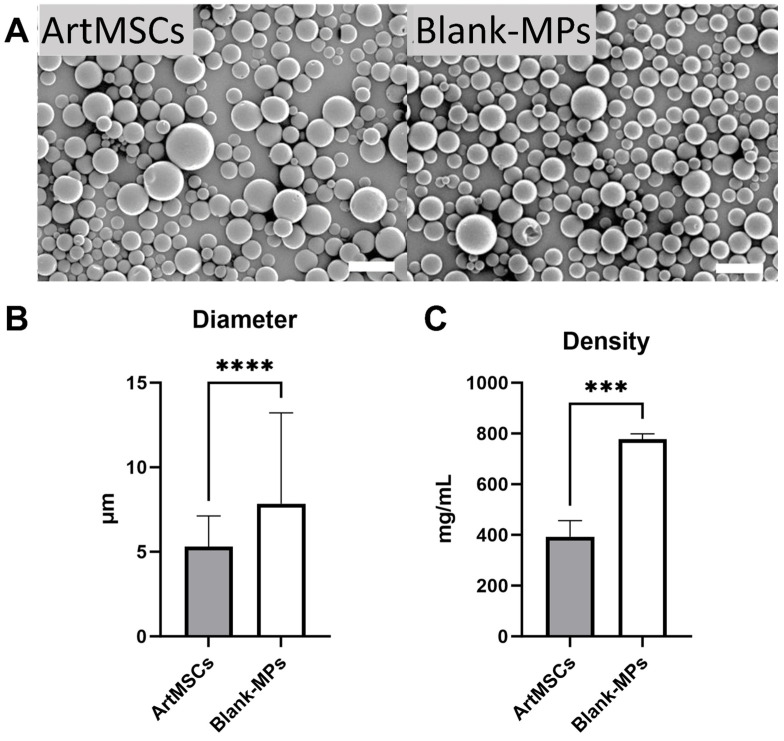
**Both microparticle types are uniform in size and non-porous** (**A**) **and ArtMSCs are smaller and less dense than Blank-MPs** (**B**,**C**). ArtMSCs (**A**, left) and Blank-MPs (**A**, right) were imaged using scanning electron microscopy. Each type of MP demonstrated relatively uniform sizes and non-porous morphology, with no noticeable qualitative difference between groups. Scale bar = 10 µm. ArtMSCs had a smaller average diameter (**B**) and were uniform in diameter compared to Blank-MPs. ArtMSCs also had a smaller average density (**C**) compared to Blank-MPs. *** = *p* < 0.001, **** = *p* < 0.0001.

The ArtMSC and Blank-MP morphologies are shown in Figure 2A, demonstrating relatively uniform spherical shapes of microspheres in both groups. However, the diameters of the ArtMSCs were 5.31 ± 1.81 µm and the Blank-MPs were 7.85 ± 5.37 µm (n = 10,000 MPs per sample, *p* < 0.0001). Additionally, the ArtMSCs had a density of 393.3 ± 63.0 mg/mL while the Blank-MPs had a density of 776.9 ± 21.6 mg/mL (n = 3, *p* = 0.0006). Thus, the ArtMSCs were smaller and less dense than the Blank-MPs, as shown in Figure 2B,C.

### 3.2. Protein Release from ArtMSCs

**Figure 3:**
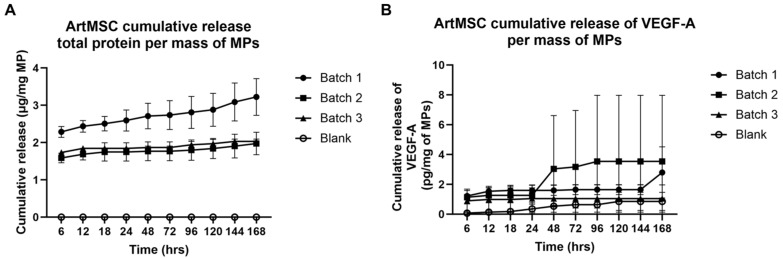
**Burst release of total protein and VEGF-A was observed with ArtMSCs.** ArtMSCs showed a release of protein within the first 6 h of incubation in all three batches, with no protein detected in the Blank-MPs at this time point (**A**). After the burst release, little to no protein was released from any ArtMSC batches. ArtMSCs also demonstrated a release of VEGF-A within the first 6 h of incubation in all three batches (**B**). VEGF-A release was minimal from Blank-MPs. Following burst release, there was little to no additional VEGF-A detected in the releasates.

An initial burst release of protein cargo was observed within the first 6 h of incubation of the ArtMSCs (Batch 1: 2.287 ± 0.146 µg/mg of MPs, *p* = 0.0027, Batch 2: 1.587 ± 0.127 µg/mg of MPs, *p* = 0.0053, and Batch 3: 1.735 ± 0.059 µg/mg of MPs, *p* < 0.0001) compared to Blank-MPs with no burst protein release (0.000 ± 0.000 µg/mg of MPs) (Figure 3A). This burst release contributed to anywhere from 64.79 to 89.21% of total protein released. VEGF-A was detected within this burst release of protein cargo (Batch 1: 1.217 ± 0.142 µg/mg of MPs, *p* = 0.0017, Batch 2: 1.137 ± 0.453 µg/mg of MPs, *p* = 0.1209, and Batch 3: 0.897 ± 0.778 µg/mg of MPs, *p* = 0.4520) (Figure 3B). Only the Batch 1 release had significant levels of VEGF-A detected, and burst release contribution was more variable, ranging from 22% to 100% of total VEGF-A released over the 7-day period. There was no detectable uPA in any ArtMSC releasates and no distinguishable difference in uPA activity in the CM compared to the nCM (Figure S1).

### 3.3. In Vitro Analysis of CM and ArtMSCs

**Figure 4:**
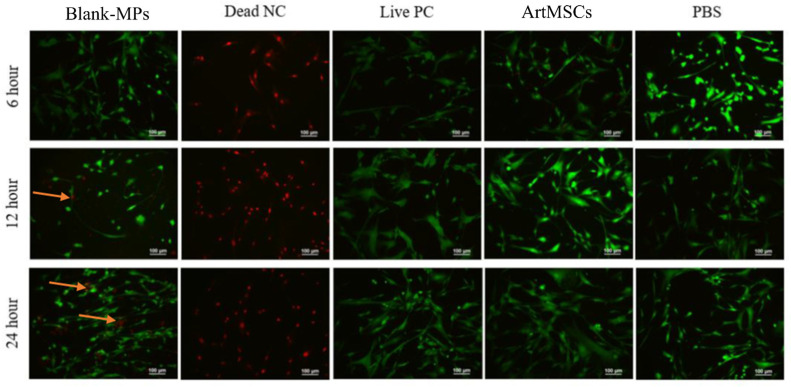
**ArtMSCs did not induce SMC toxicity.** ArtMSCs and Blank-MPs were incubated with SMCs for 6, 12, and 24 h, after which the SMCs were incubated with a fluorescent LIVE/DEAD assay. Green cells indicate live cells while red cells indicate dead ones. Blank-MPs caused some cytotoxicity at 12 h and 24 h, as indicated by the red cells shown by the orange arrows. The cell death control (Dead NC using diH_2_O) and live cell control (Live PC using SBM) both indicate that the LIVE/DEAD stain was functional.

The ArtMSC releasates did not demonstrate cytotoxicity to cultured SMCs while Blank-MP releasates showed some cytotoxicity after 24 h in culture (Figure 4). At 6 h, ArtMSC releasates and Blank-MP releasates had comparable numbers of live cells (75 cells to 74 cells) and dead cells (5 cells to 2 cells). However, while ArtMSC releasates had relatively unchanged numbers of live and dead cells at 12 h (57 live cells and 1 dead cell) and 24 h (73 live cells and 1 dead cell) compared to 6 h, Blank-MP releasates had more dead cells at 12 h (42 live cells and 14 dead cells) and 24 h (99 live cells and 47 dead cells) compared to that at 6 h. The positive control (SBM) had relatively unchanged numbers of cells over time (6 h: 55 live cells, 0 dead cells; 12 h: 57 live cells, 1 dead cell; 24 h: 80 live cells, 2 dead cells); the negative control appeared to have relatively no change in live cells and dead cells over time as well (6 h: 1 live cell, 25 dead cells; 12 h: 0 live cells, 59 dead cells; 24 h: 0 live cells, 49 dead cells). PBS also had similar numbers of live and dead cells over time (6 h: 61 live cells, 0 dead cells; 12 h: 62 live cells, 0 dead cells; 24 h: 77 live cells, 0 dead cells).

**Figure 5:**
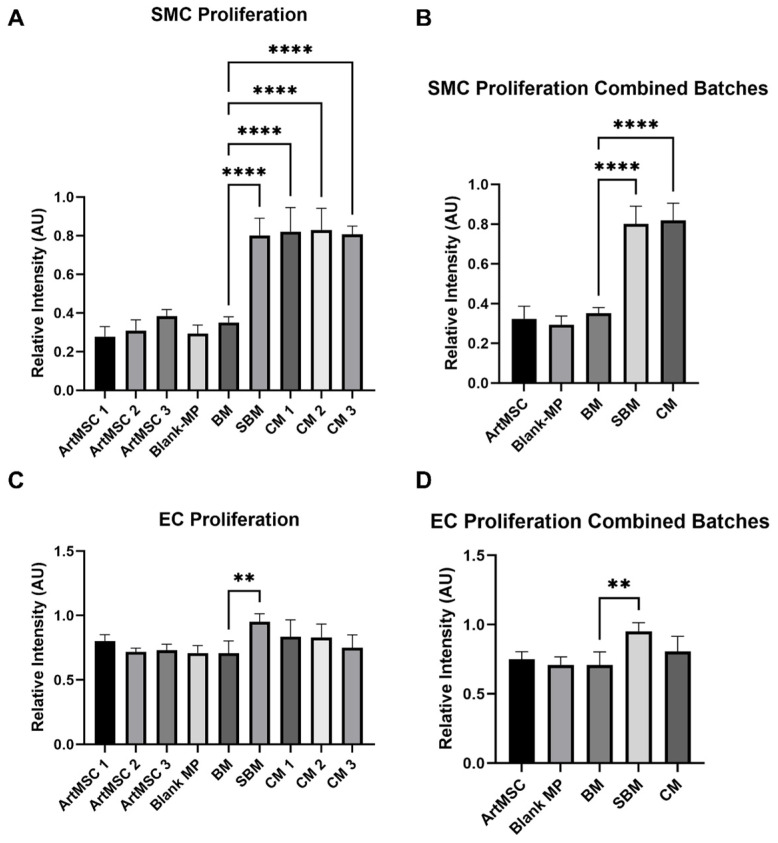
**CM, but not ArtMSC releasates, induced SMC proliferation but not EC proliferation.** CM induced significantly more proliferation in SMCs, while ArtMSCs did not increase proliferation compared to BM. Neither CM nor ArtMSCs increased proliferation of ECs. SMC proliferation of all three different batches of ArtMSCs, their respective CM, Blank-MPs (negative control), BM (negative control), and SBM (positive control) (**A**) are shown, along with combining the SMC proliferation data for all batches with the aforementioned controls (**B**). EC proliferation of these same batches of ArtMSCs, CM, and similar controls for SMCs (**C**) are also shown, along with combining the EC proliferation for all batches (**D**). ** = *p* < 0.01, **** = *p* < 0.0001.

The ArtMSC releasates did not increase SMC proliferation following 24 h of culture (Figure 5A,B) compared to BM. The CM for all three batches resulted in more SMC proliferation (Batch 1: 0.821 ± 0.124 AU, *p* < 0.0001, Batch 2: 0.829 ± 0.112 AU, *p* < 0.0001, Batch 3: 0.807 ± 0.042 AU, *p* < 0.0001) compared to BM (0.352 ± 0.029 AU) (Figure 5A). Also, combining all three batches of CM (0.819 ± 0.087 AU, *p* < 0.0001) demonstrated overall greater SMC proliferation compared to BM (Figure 5B). The CM did not change EC proliferation (Figure 5C,D); these results are also outlined in Table S1.

### 3.4. Explant Analysis of ArtMSC Lyogel TEVGs

**Figure 6:**
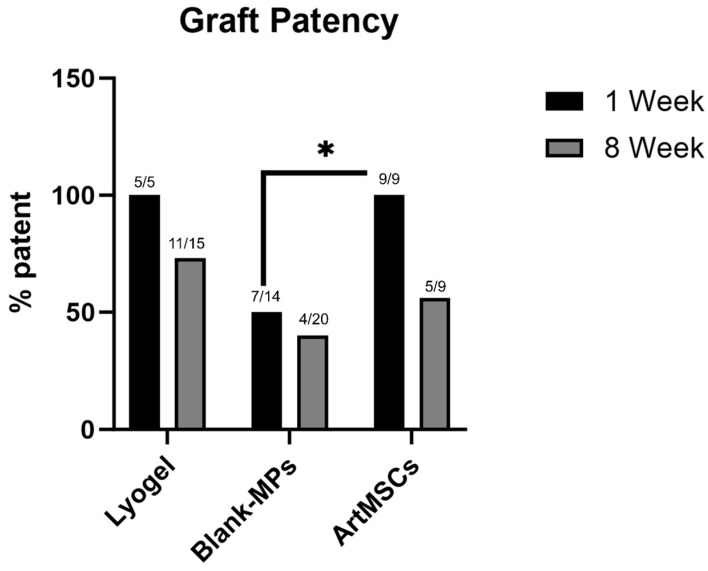
**ArtMSC-loaded grafts promoted acute patency at 1 week.** In 1-week grafts, ArtMSC-loaded grafts had significantly higher patency (100% (5/5)) compared to Blank-MP grafts (50% (7/14)). There was a trend toward a decrease in patency at 8 weeks for all groups compared to week 1, but no difference in patency across groups. * = *p* < 0.05.

The patency rate of the ArtMSC lyogel TEVGs was 100% (9/9) at 1 week. In comparison, unloaded and Blank-MP lyogel constructs had patency rates of 100% (5/5) and 50% (7/14), respectively, at 1 week as previously reported [10]. At 8 weeks, the patency rate of the ArtMSC lyogel TEVGs was 56% (5/9) compared to unloaded and Blank-MP lyogel constructs with patency rates of 73% (11/15) and 40% (8/20), respectively, as previously reported [10]. The overall results are shown in Figure 6. There was a trend toward lower patency in all 8-week explant groups compared to 1 week, while there was a higher patency of 1-week ArtMSC grafts compared to 1-week Blank-MP grafts (*p* = 0.0189). All macroscopic images are representative of the various explants with green or red borders indicating patency determined via angiography (Figures S2–S5). The ArtMSC grafts had little to no signs of stenosis at 1 week (Figure S2). Blank-MP grafts at 1 week had clear stenosis and occlusion as early as 1 day post implantation (Figure S3). The ArtMSC explants at 8 weeks demonstrated stenosis in 4 of the 5 patent implants. (Figure S4). The Blank-MP explants at 8 weeks also had stenosis in patent implants and clear occlusion in other grafts (Figure S5).

**Figure 7:**
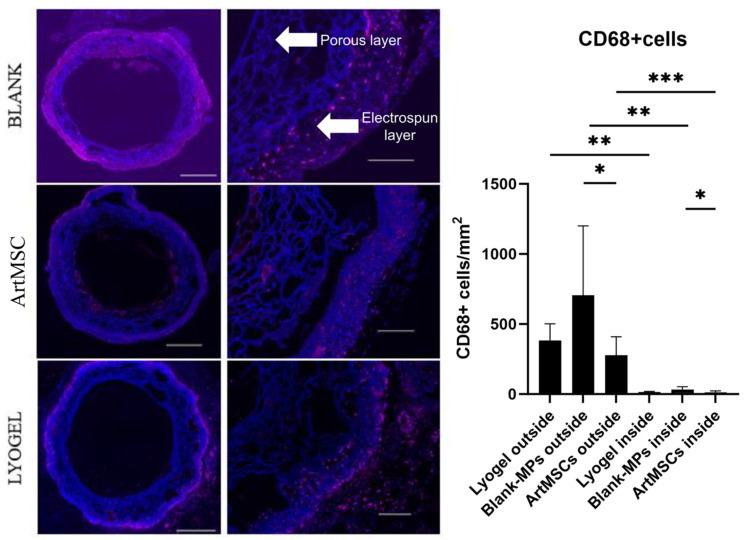
**ArtMSC-loaded grafts reduced the macrophage response at 1 week.** ArtMSC-seeded grafts (n = 9) had significantly lower numbers of CD68+ macrophages in both layers compared to Blank-MP seeded grafts (n = 11). Whole cross sections (left, scale bar = 500 µm) for each graft type were stitched together from individual images (one from each is presented on the right, scale bar = 100 µm). All cell nuclei were stained with bisbenzimide (blue), and macrophages were stained for CD68+ marker (pink). The number of CD68+ cells in both the outer electrospun layer (outside for each graft type) and inner porous layer (inside for each graft type) are shown on the graph on the righthand side. The outer electrospun layer of the grafts also had significantly higher numbers of macrophages compared to the inner porous layer for all three graft types, indicated by the pink coloration in the stained images and shown numerically in the graph of number of CD68+ cells. * = *p* < 0.05, ** = *p* < 0.01, *** = *p* < 0.001.

In Figure 7, two distinct layers of 1-week explants are shown: the inner porous layer and the outer electrospun layer. There was a higher concentration of macrophages (indicated by pink coloration in Figure 7) in the outer electrospun layer of the graft compared to the inner porous layer in all three groups: ArtMSCs (n = 9) (outside: 277.4 ± 132.2 vs. inside: 13.1 ± 10.5 CD68+ cells/mm^2^, *p* = 0.0003), lyogel (n = 4) (outside: 382.8 ± 119.4 vs. inside: 14.5 ± 4.4 CD68+ cells/mm^2^, *p* = 0.0228), and Blank-MPs (n = 11) (outside: 704.9 ± 495.1 vs. inside: 33.4 ± 20.3 CD68+ cells/mm^2^, *p* = 0.0004) (Figure 7). There was a significantly lower number of macrophages on both the outside (*p* = 0.0355) and inside (*p* = 0.0222) regions of the ArtMSC grafts compared to the Blank-MP grafts.

**Figure 8:**
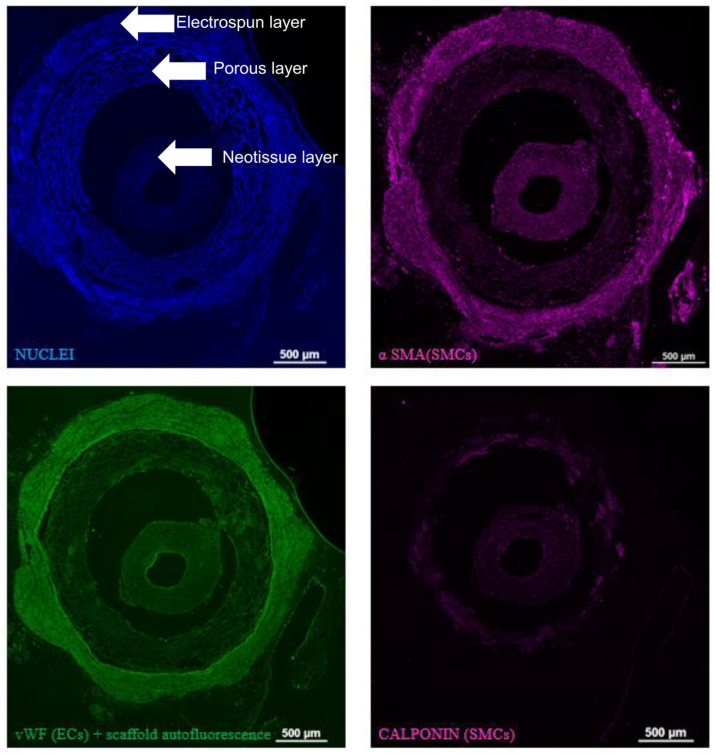
**ArtMSC grafts recruited contractile smooth muscle cells within the electrospun and neotissue layers and endothelial cells in the neotissue layer.** Full cross section of a representative ArtMSC graft stained with IFC indicated SMC (two right images, pink) and EC (bottom left, green) presence in the inner neotissue layer and electrospun layer (outer layer), but not within the porous layer (middle layer). Cell nuclei (blue) were also stained with bisbenzimide (upper left). Some delamination between the individual layers is also seen. Scale bar = 500 µm.

**Figure 9:**
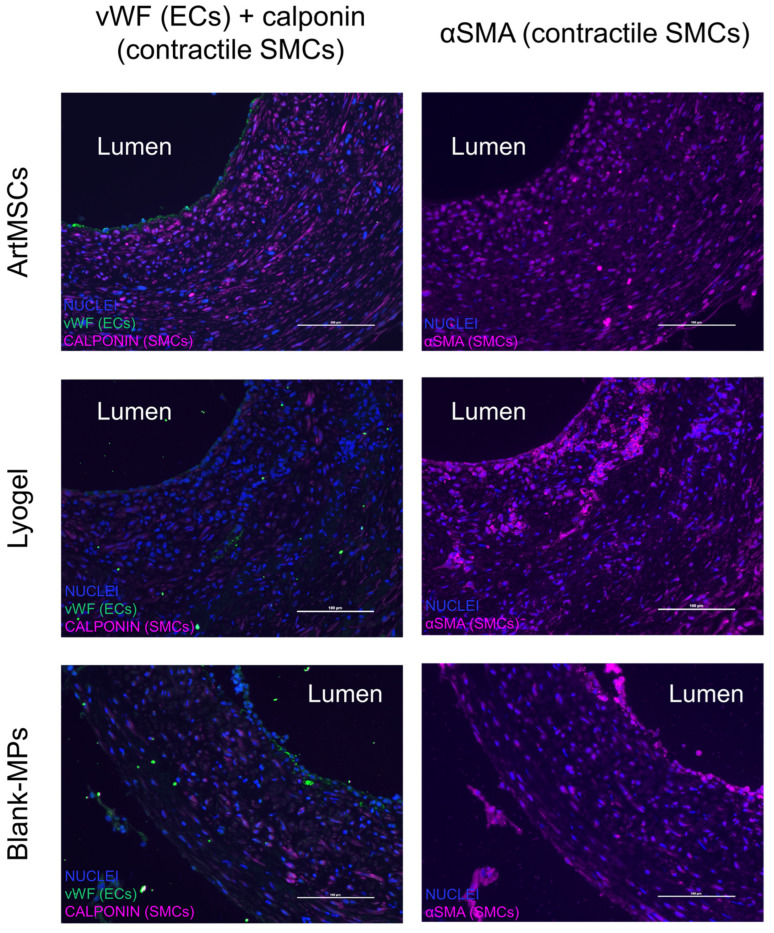
**ArtMSC grafts promoted the recruitment of contractile smooth muscle cells and endothelial cells within the inner neotissue layer of TEVGs.** Comparison of immunofluorescent staining between a representative ArtMSC explant and representative unloaded lyogel and Blank-MP-loaded explants at 8 weeks showed higher signal of contractile SMCs stained with calponin (first column, pink) and α-SMA (second column, pink) within the inner most tissue layer. An endothelial lining was also detected using vWF (first column, green), indicating endothelial incorporation into the ArtMSC grafts but not as strongly in the controls. Scale bar = 100 µm.

**Figure 10:**
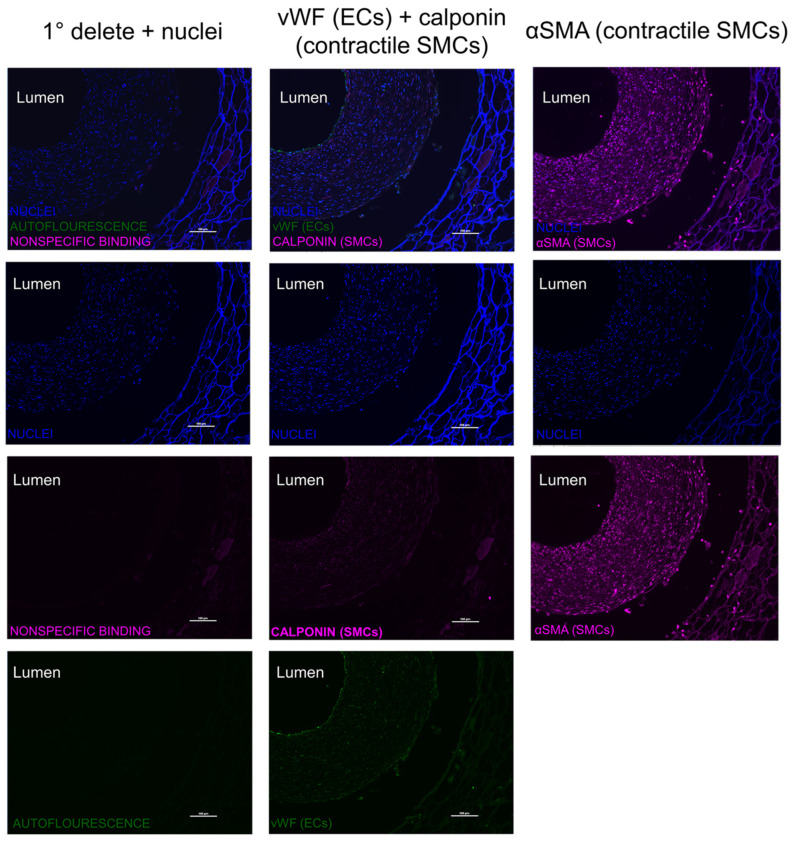
**The majority of cells within the neotissue layer of ArtMSCs were contractile smooth muscle cells, and a thin inner endothelial layer was also present.** Immunofluorescent staining of a representative ArtMSC explant at 8 weeks demonstrated contractile SMCs stained with calponin (second column, pink) and α-SMA (third column, pink) within the inner most tissue layer. The endothelial lining was also stained using vWF (second column, green), indicating endothelial incorporation into the graft. The first column was used as a primary delete negative control for the ArtMSC explants, indicating any potential background staining. This image separated the different color channels to see each antibody stain individually. The inner neotissue layer of the explant contained the majority of the incorporated cells vs. the porous layer of the graft (bottom right of each image) which was relatively acellular. Scale bar = 100 µm.

**Figure 11:**
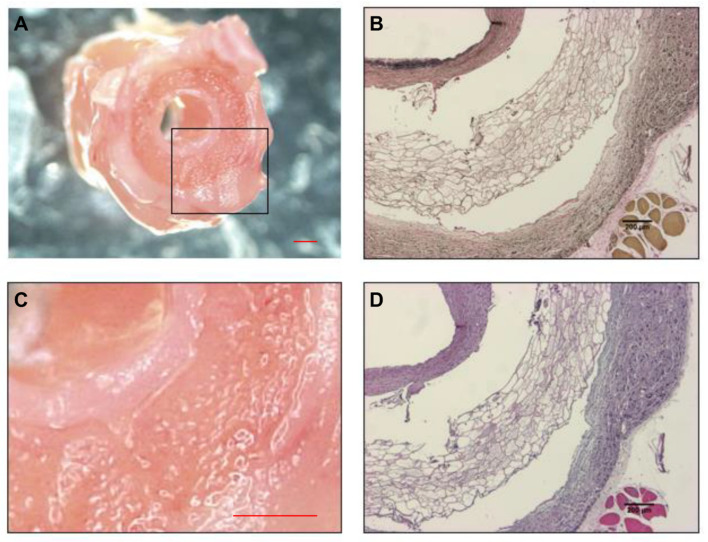
Histological staining demonstrated cell recruitment to the neotissue layer and electrospun layer and matrix deposition in 8-week explants. IHC of a representative ArtMSC graft showed cellularity within the different layers of the graft at 8 weeks. Macroscopic neotissue formation is grossly shown (**A**) with a closer image of the region enclosed by the black box as panel (**C**). VVG staining (**B**) also showed some initial matrix deposition, indicated by the purple stain in the inner neotissue layer. H&E staining (**D**) showed cellular infiltration into the inner neotissue layer and outer electrospun layer, shown by the darker purple coloration. Scale bar (black for (**B**,**D**)) = 200 µm and scale bar (red for (**A**,**C**)) = 1000 µm.

As indicated in Figure 8, three distinct layers of 8-week explants can be seen: the inner neotissue layer, middle porous layer, and outer electrospun layer. When comparing between representative IFC images of the 8-week explants, the ArtMSC grafts demonstrated higher fluorescent signal of calponin and α-SMA compared to the lyogel and Blank-MP grafts, indicating a larger presence of contractile SMCs. Some minor delamination occurred between the various layers of the graft, but this is most likely an artifact of processing the grafts. This delamination has been seen previously in both silk grafts and other grafts from our group [7,8]. Delamination of TEVGs has also been reported by other groups [42,43,44]. The ArtMSC grafts also demonstrated a higher fluorescent signal of vWF than the controls, indicating a larger presence of ECs (Figure 9). At 8 weeks, IFC images demonstrated the presence of α-SMA and calponin, indicating contractile SMCs and vWF, indicating ECs within the neotissue layer of the ArtMSC grafts (Figure 10); this representative image also showed some stenosis. Figure 10 separates out the different colored channels to see the signal of different stains. The majority of cells were found in the neotissue layer and electrospun layer. This cellularity was further confirmed by hematoxylin and eosin (H&E) staining while Verhoff–Van Gieson (VVG) staining demonstrated early ECM formation within the neotissue layer (Figure 11).

## 4. Discussion

There were some observed differences between the ArtMSCs and Blank-MPs. The ArtMSCs were less dense compared to the Blank-MPs, most likely due to the higher salt content of the CM compared to the ultrapure water loaded into the Blank-MPs. One potential explanation for the decreased density would be an increased internal porosity of the ArtMSCs, but the presence of these internal pores was not validated. The CM from the MSCs was successfully encapsulated within PLGA microparticles and exhibited a burst release of 1.870 ± 0.335 µg/mg of protein within the first 6 h of incubation. While the majority of protein was released within the first 6 h, not all of the PLGA particles were completely degraded, as PLGA particles take longer than one week to completely degrade [45]. VEGF-A was detected in the initial burst of the ArtMSC releasate while uPA was not detected in any releasates. uPA activity was also not detected in the CM compared to the nCM, implying that functional uPA was not produced by the MSCs during conditioning. These results contrast with our previous findings [9] where MSCs produced uPA in their CM. This difference in uPA production could be attributed to the cell type and basal media used. In this current study, commercially sourced RoosterBio MSCs were used along with the company’s supplemented media to produce the CM while our previous study [9] utilized primary patient-sourced adipose-derived MSCs and a different media supplemented with fetal bovine serum. While protein and VEGF-A were detected in the releasates, sufficient encapsulation of VEGF-A and other cytokines that affect vascular cell activity may not have been achieved.

There was no additional proliferative effect of the ArtMSCs compared to unsupplemented media (negative control), while the CM had an increased proliferative effect similar to that of supplemented media (positive control). This could potentially be due to poor CM loading into the ArtMSCs or ineffective release of the CM from them.

It appears that there is a negative effect of the PLGA microparticles themselves in vivo, as the effect was initially offset by the inclusion of CM at 1 week. However, this offsetting effect is not as potent after 8 weeks. This stenotic tissue growth may have resulted from components of the CM secretome overstimulating the surrounding endothelium, resulting in excessive tissue growth.

The success of a TEVG relies upon the initial recruitment of immune cells to the graft to initiate host cell infiltration followed by a proliferative healing phase in which the scaffold itself is remodeled into vascular tissue [46,47,48]. These two distinct phases can be characterized by the type of macrophages present within the graft. Macrophages can be polarized toward inflammatory M1 phenotypes (typically recruited within the first 3–7 days) or regenerative/anti-inflammatory M2 phenotypes (polarized around 5–10 days post-implantation) [47,49,50,51,52]. In this study, macrophages were recruited more heavily to the Blank-MP-loaded grafts both inside and outside compared to the ArtMSCs. While the initial recruitment appeared to occur, the subsequent polarization toward M2 phenotypes may not have occurred, resulting in prolonged inflammation and excessive tissue growth leading to stenosis. The higher concentration of macrophages in the outer layers of the grafts suggested larger recruitment of resident macrophages compared to circulating monocytes within the blood. The nanofibrous nature of the electrospun layer could also play a role in this macrophage recruitment, as others have indicated the ability of nanofibrous material to recruit and polarize macrophages in vivo [53,54,55].

ECs are necessary for antithrombotic properties while SMCs can deposit new ECM and other blood vessel constituents important for overall remodeling. At 8 weeks, both ECs and SMCs were able to form a layer of new vascular tissue along the lumen of the graft in addition to some small infiltration into the porous layer. This increased migration and sustained presence within the outer ES coating in all three groups may also be caused by difference in porosity between two layers.

In our previous work, we seeded a variety of cellular [8,9,11] and acellular [6,10] cargo into TEVG scaffolds, including CCL2-loaded MPs used to generate seeded lyogel scaffolds [10]. With CCL2, patency within both the first week and within eight weeks was similar to those achieved using ArtMSCs within this study. Recruitment of macrophages both in the inside and outside layer of the grafts was less in the ArtMSC grafts compared to the CCL2 MP grafts. As CCL2 is a specific chemoattractant for macrophages, it is reasonable that more macrophages were recruited to the CCL2 graft.

MSCs seeded into polyester urethane urea scaffolds were another previous graft configuration that yielded promising results. MSCs from healthy donors provided 100% patency at 8 weeks [11], which was a key rationale for utilizing MSC-based CM for the development of artificial MSCs. Our lab has utilized EVs isolated from CM to seed into silk-based grafts [6]. These grafts also had 100% patency at 8 weeks, demonstrating both the regenerative potential of silk and factors contained in the CM.

Other groups have evaluated cellular-based cargo in TEVGs. Roh et al. embedded CCL2-containing alginate particles into their grafts to replicate its secretion from bone marrow mononuclear cells (BMCs), similar to how we encapsulated CM to replicate secretion of regenerative cytokines from MSCs [56]. Additionally, all of their CCL2 particle grafts at 10 weeks were patented and were histologically similar to those seeded with BMCs. Another group using silk-based TEVGs also saw macrophages heavily recruited to the outer layer of their grafts, indicating that silk-based grafts may have a larger effect on resident macrophages rather than circulating monocytes [57].

Mohammadi et al. implemented a controlled release system of CM to reduce the initial inflammatory response and reduce the overall chance of rejection of implanted materials [58]. Similar to our PLGA ArtMSCs, they loaded umbilical cord MSC-based CM into alginate capsules. Both our study and Mohammadi et al. showed a release of CM from our delivery systems, but they investigated the release for only 48 h rather than 1 week. They found that their CM-containing capsules reduced inflammatory activity of macrophages in vitro through the reduction in CD68 activation. In vivo, they subcutaneously implanted the alginate capsules with non-conditioned media and conditioned media and found reduced numbers of CD68+ cells, similar to our ArtMSC-loaded grafts having reduced CD68+ cells compared to Blank-MP controls. Again, this study solely looked at the short-term effects of the CM-loaded capsules implanted subcutaneously in mice, compared to our study where we investigated arterial interposition grafts in rats at short- and long-term time points.

There are some limitations regarding the utilization of ArtMSCs versus cellular delivery. Cells within a graft can engage in dynamic reciprocity [59] with their environment to potentially provide anti-inflammatory and regenerative cytokines whereas ArtMSCs are unable to adapt to a changing environment. Once the MPs encapsulate desired cargo, that cargo cannot easily be altered. With these experiments, loading of protein cargo was observed. However, there was no optimization of CM loading into the PLGA MPs nor optimization of ArtMSC loading into lyogel scaffolds. There was no analysis of protein release from the ArtMSC-loaded lyogel scaffolds, though given our previous results with CCL2-loaded lyogel scaffolds [10], we expect similar results (burst release of cargo within 24 h) with the ArtMSC-loaded scaffolds. Additionally, during the fabrication process, the loaded scaffolds were soaked in 80% ethanol, which could have potentially caused premature burst release of active proteins prior to implantation. However, our previous CCL2-loaded lyogel scaffolds were fabricated in a similar manner and still exhibited burst release of CCL2 [10]. To see potentially more regenerative effects on cellular activity both in vitro and in vivo, the fabrication and loading processes need further optimization. For example, changing the kind of PLGA [33] and ratio of lactic acid to glycolic acid could change the release profile, which could in theory affect the patency at later time points [60]. The CM itself is not well characterized and contains FBS and various other factors that can unintentionally affect vascular and immune cell activity. Some other cytokines that could be contained in the CM include fibroblast growth factor 2, hepatocyte growth factor, and chemokine ligand 12, but these were not analyzed in this study [33,61]. Additional analysis of the components of the CM, experiments with these components that show their effects on cellular activity, and investigation into serum-free media alternatives should be considered in future studies.

Some limitations were present for our in vitro assays. Our proliferation assays did not investigate non-conditioned media control. If there were stimulating factors within the MSC growth media itself, that could have also played a role in the proliferation of the SMCs. Insufficient loading of the CM into the ArtMSCs was most likely the cause for no additional SMC proliferation, but this was not investigated fully. Furthermore, there was little to no release following the initial 6 h of release. As the other regenerative processes may need to be activated later for improved regeneration, sustained release of the CM needs to be improved. In our cytotoxicity assays, cell viability could be highly compromised since the cells were incubated for 12 h in PBS, though we did not see this issue in our images. Additionally, in analysis of the cell counts for these cytotoxicity assays, counts were only performed on one well and thus statistical analysis could not be performed. The manner in which the CM was cultured was not changed from standard culturing protocols. Other groups [62,63,64] have found that changing culture conditions such as hypoxia can alter components in CM, which is another direction for optimization of this work.

Another limitation of this study was only looking at general macrophage recruitment in TEVG explants rather than polarization of macrophages toward M1 or M2 phenotypes, as both types play a role in the remodeling of TEVGs [47,49,50,51,52]. Additional staining for M1 and M2 markers should be performed to have a better assessment of macrophage-driven TEVG remodeling. Only two time points were assessed to look at acute and longer-term patency and remodeling of the ArtMSC TEVGs. As other groups [46,48,65] have investigated shorter and longer time points to see the various stages of remodeling of their TEVGs, we can also look into these other time points to have a better understanding of how our cargo affects tissue regeneration and remodeling. For example, having a timepoint between the first 3–7 days to investigate early M1 markers and another timepoint between 5–10 for M2 markers, as indicated earlier, could potentially capture a better picture of the remodeling of our grafts.

Our rat model also did not investigate any potential foreign body response caused by the CM or the PLGA microspheres. As PLGA hydrolytically degrades into lactic and glycolic acid, it could also elicit an immune response [66,67]. While macrophages play a role in this response, other cells such as neutrophils are also key players [47,68]. Our study only looked at macrophage recruitment, which addresses the innate immune response. However, the adaptive immune response, including T cells and natural killer cells, also plays a role in the remodeling of the TEVG [47,68]. Gu et al. [67] saw inflammation from a PLGA-containing implant; this response was then attenuated by dexamethasone, similar to how our CM reduced the acute immune response caused by PLGA. Investigating the responses of other immune cells should be taken into consideration for future studies. Additionally, as the PLGA degrades and more cells degrade the scaffold, together they could potentially affect the compliance of the TEVG [69]. We did not measure the mechanical properties of our explants and thus did not determine if there was a compliance mismatch. However, we expect that the mechanical properties of the ArtMSC TEVGs would be similar to those measured in our previous publication [10]. In future studies, we should also look at how the compliance changes overtime with our TEVGs to ensure compliance mismatch is not occurring.

Using MSCs in clinical treatments has limitations including short viability and retention of transplanted MSCs in vivo [70,71], the requirement for high doses of cells (typically achieved through large-scale culturing), and the potential need for repeated treatments. Additionally, our lab has shown that autologous MSCs from high risk cardiovascular populations are less able to prevent thrombosis [9]. Cryopreservation of MSCs for later use can lower the therapeutic efficacy and overall viability of the cells [72]. Using cell-free products containing the same paracrine factors secreted by MSCs can alleviate these issues. Creating a sustained release of the paracrine factors can provide a more efficacious treatment compared to MSCs that do not remain viable for longer periods of time. While the controlled release products cannot be altered following encapsulation, they may provide an advantage over MSCs, as ArtMSCs cannot differentiate to other cell lineages or respond to their environment by secreting potentially harmful cytokines in response to the harsh environment. From a regulatory perspective, cell-free products can more easily be translated to the clinic and eventual commercial use.

## 5. Conclusions

CM from the MSCs was able to be encapsulated into and released from PLGA-MPs, creating “artificial MSCs”. In vitro analysis of the ArtMSCs revealed an initial burst release of protein containing VEGF-A but a reduction in regenerative activity compared to CM. In vivo, acute 1-week patency was achieved in ArtMSC TEVGs while patency was lower in 8-week explants. Macrophage recruitment at 1 week and stenosis at 8 weeks was also observed with the ArtMSC TEVGs. Additional optimization of CM encapsulation and retention of regenerative capabilities is needed, but this work provides a promising foundation for creating a cell-free regenerative TEVG.

## 6. Patents

The technology used in this research is patent pending, titled “Artificial Cells and Delivery Devices for Use in Tissue Engineering, and Related Methods”, Serial No.: 16/308,889.

## Data Availability

The data presented in this study are available on request from the corresponding author.

## Supplementary Materials

The following supporting information can be downloaded at: https://www.mdpi.com/article/10.3390/bioengineering11090947/s1, Table S1. Relative proliferation of SMC and ECs following treatment with ArtMSC releasates, Blank-MP releasates, BM, SBM, or CM (mean ± STD); Figure S1. CM compared to nCM did not show active uPA activity; Figure S2. Representative gross cross sections of the ArtMSC grafts were patent after 1 week; Figure S3. Representative gross cross sections of the Blank-MP grafts demonstrated some occlusion at 1 week; Figure S4. Representative gross cross sections of the ArtMSC grafts at 8 weeks showed stenosis with lower patency than seen at 1 week; Figure S5. Representative gross cross sections of the Blank-MP grafts at 8 weeks also demonstrated stenosis and lower patency than seen at 1 week.
