# Supplementary material for: Mesenchymal Stem Cell-Conditioned Media-Loaded Microparticles Enhance Acute Patency in Silk-Based Vascular Grafts
Source: Bioengineering (Basel). 2024 Sep 21;11(9):947. doi: 10.3390/bioengineering11090947 (PMC11428691; doi:10.3390/bioengineering11090947)
Supplement: Supplementary file 1 [file bioengineering-11-00947-s001.zip › bioengineering-3104305-supplementary.pdf]

Table S1. Relative proliferation of SMC and ECs following treatment with ArtMSC releasates, Blank-MP releasates, BM, SBM, or CM (mean ± STD).

Treatment group	SMC proliferation (Relative intensity)	EC proliferation (Relative intensity)
ArtMSC 1	0.278 ± 0.051	0.802 ± 0.051
ArtMSC 2	0.310 ± 0.055	0.718 ± 0.029
ArtMSC 3	0.384 ± 0.034	0.731 ± 0.045
ArtMSC combined	0.324 ± 0.063	0.750 ± 0.054
Blank-MP	0.293 ± 0.044	0.710 ± 0.057
BM	0.352 ± 0.029	0.708 ± 0.096
SBM	0.801 ± 0.089	0.952 ± 0.063
CM 1	0.821 ± 0.124	0.836 ± 0.132
CM 2	0.829 ± 0.112	0.829 ± 0.105
CM 3	0.807 ± 0.042	0.751 ± 0.100
CM combined	0.819 ± 0.087	0.805 ± 0.110

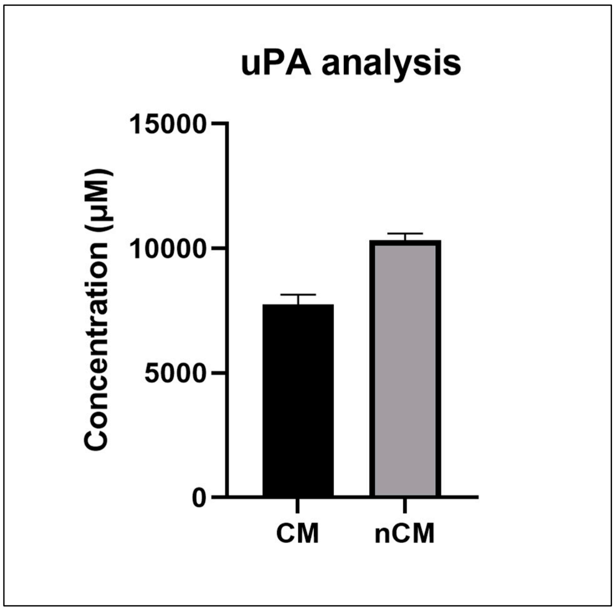

Figure S1. CM compared to nCM did not show active uPA activity. CM did not demonstrate uPA activity compared to nCM, indicating that these cells did not produce active uPA after conditioning.

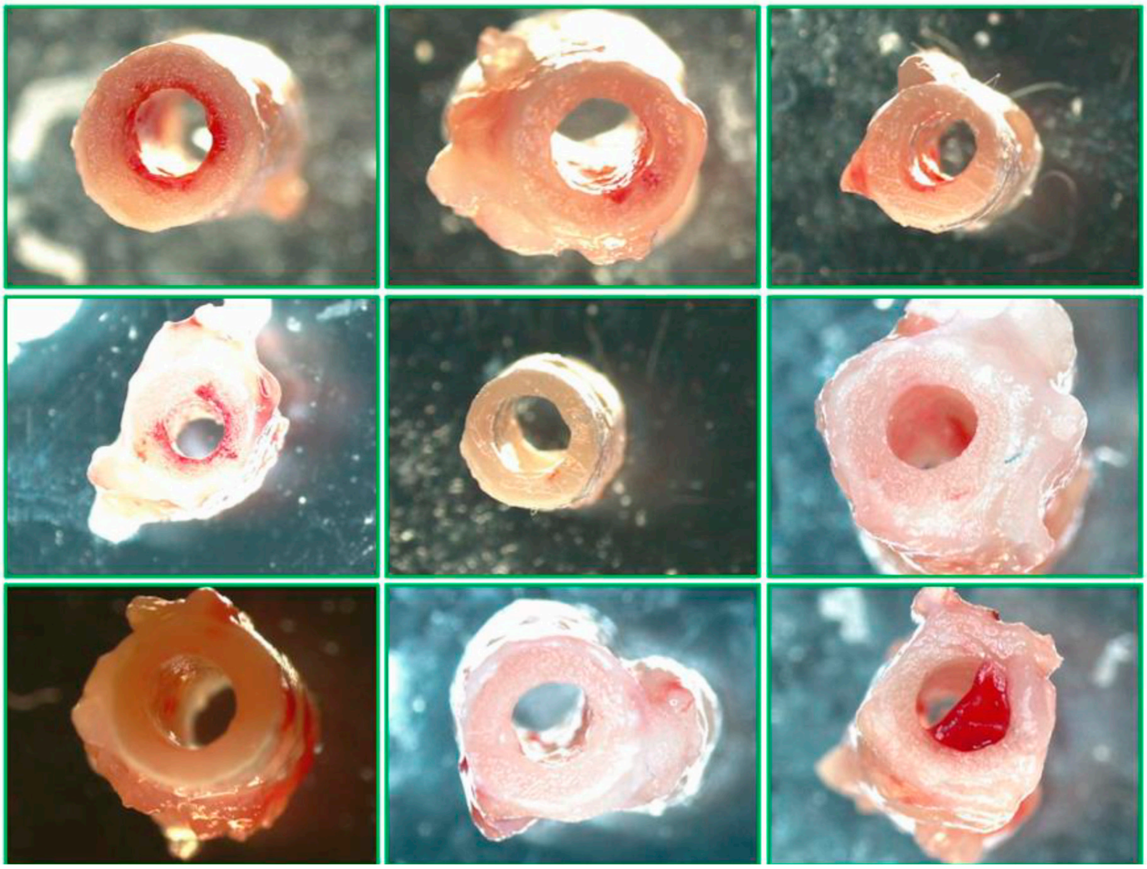

Figure S2. Representative gross cross sections of the ArtMSC grafts were patent after 1 week. Patency (green border) was observed in all 9 ArtMSC grafts at 1 week with angiography, with no sign of stenosis as shown by the open lumen in the explanted grafts.

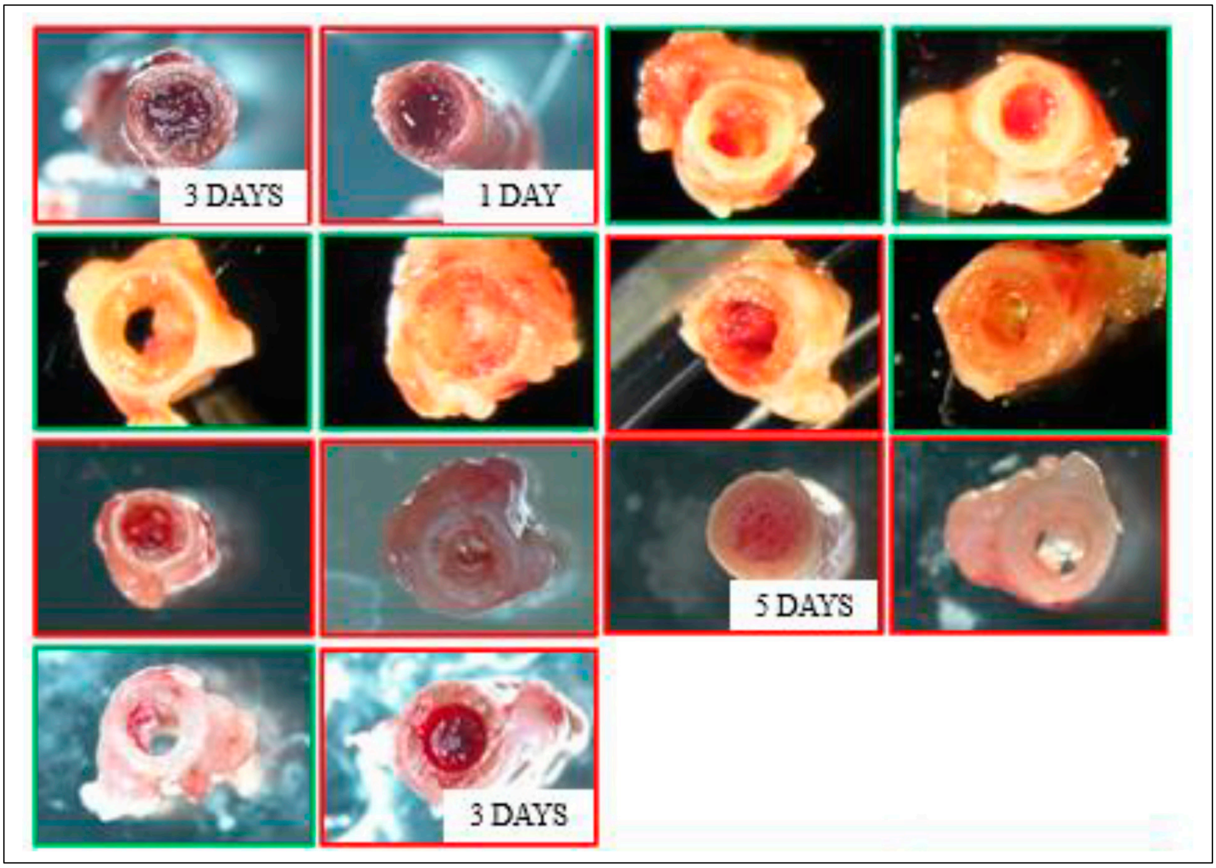

Figure S3. Representative gross cross sections of the Blank-MP grafts demonstrated some occlusion at 1 week. Blank-MP grafts at 1 week had 50% patency (green border) with angiography and showed some occlusion occurring earlier than the 1 week timepoint.

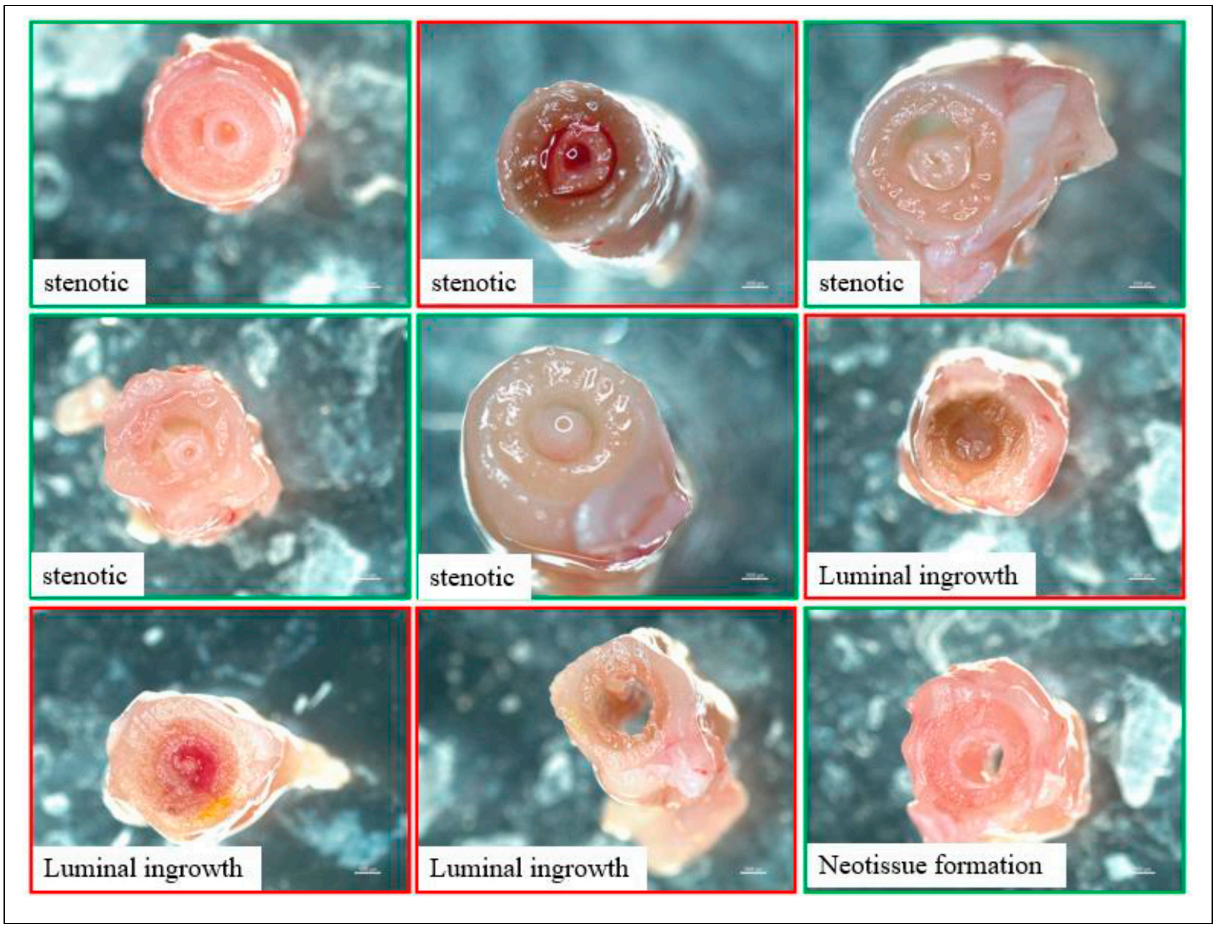

Figure S4. Representative gross cross sections of the ArtMSC grafts at 8 weeks showed stenosis with lower patency than seen at 1 week. ArtMSC grafts at 8 weeks had 56% patency (green border) with angiography and also demonstrated some stenosis. Luminal ingrowth was also observed in explants. Stenosis was observed in occluded grafts (red border) as well.

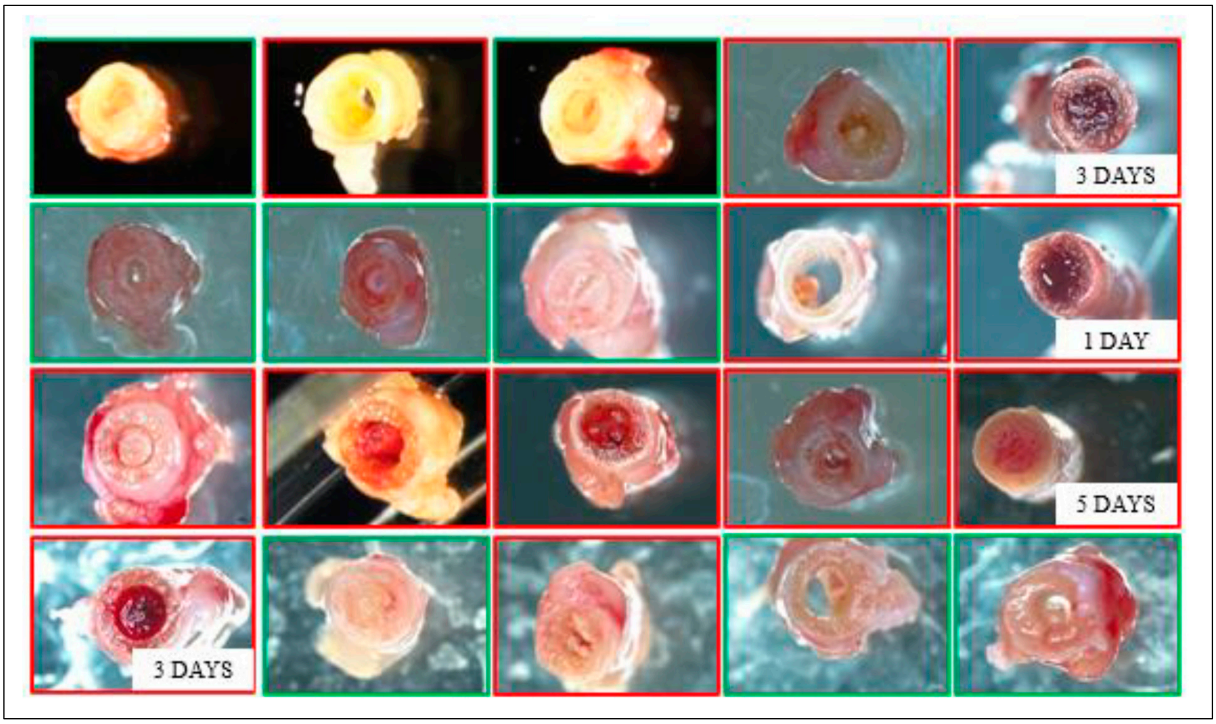

Figure S5. Representative gross cross sections of the Blank-MP grafts at 8 weeks also demonstrated stenosis and lower patency than seen at 1 week. Blank-MP grafts at 8 weeks also showed stenosis and had patency of 40% (green border) with angiography. The 1 week explants were also included in the calculations for patency and images for 8 weeks.
